# Ultrasound-based shrinkage patterns at mid-neoadjuvant chemotherapy for predicting pathological complete response in invasive breast cancer

**DOI:** 10.3389/fonc.2026.1824011

**Published:** 2026-09-03

**Authors:** Yiting Hong, Wenzhi Zheng, Hong Chen, Jingchao Chen, Ming He, Kangjian Wang, Haolin Shen

**Affiliations:** 1 Department of Ultrasound, Zhangzhou Affiliated Hospital of Fujian Medical University, Zhangzhou, Fujian, China; 2 Fujian Medical University, Fuzhou, Fujian, China; 3 Department of Ultrasound, Women and Children’s Hospital, School of Medicine, Xiamen University, Xiamen, Fujian, China

**Keywords:** invasive breast cancer, neoadjuvant chemotherapy, pathological complete response, predictive model, shrinkage pattern

## Abstract

**Objective:**

To develop an ultrasound-based tumor shrinkage pattern and subsequently construct a predictive model for pathological complete response by analyzing changes in ultrasonic features and measurements at mid-treatment of neoadjuvant chemotherapy (NAC) in invasive breast cancer, ultimately evaluating its clinical applicability.

**Methods:**

This study included all breast cancer patients who underwent NAC and subsequent surgery between January 2017 and December 2024. All patients received breast ultrasound examinations before and during NAC. All breast tumor images were reviewed by two experienced sonographers, who assessed and documented the ultrasound characteristics of the tumors. Based on the comparison of parameter changes, the tumors were classified into two patterns: centripetal shrinkage (CS) and non-centripetal shrinkage (NCS). NCS was further subdivided into four patterns: echogenic change, partial shrinkage, dendritic shrinkage, and fragmentation. According to the postoperative pathological results, patients were categorized into two groups: the pathological complete response (pCR) group and the non-pCR group. Univariate analysis and multivariate logistic regression analysis were performed to identify independent predictors for the predictive model. A nomogram was constructed, and receiver operating characteristic curves were plotted to evaluate the model’s performance.

**Results:**

The study included a total of 287 lesions. The results of the logistic shrinkage analysis indicated CS, ΔS(the rate of area change), echogenicity changes, Adler decrease, HER-2 positivity, and Ki-67 were identified as independent predictors of pCR (all P< 0.05). The nomogram model based on these factors demonstrated areas under the curve of 0.906 (95% CI = 0.866 - 0.946) in the training set and 0.850 (95%CI = 0.770 - 0.929) in the testing set. The univariate model using only the shrinkage pattern (CS vs NCS) yielded an AUC of 0.741 (95% CI: 0.680 - 0.803).

**Conclusion:**

It is feasible to establish tumor shrinkage patterns based on ultrasound examinations during the mid-NAC stage. The nomogram-based predictive model, which combines ultrasound image changes and tumor pathological indicators, can predict NAC outcomes and holds certain clinical application value.

## Introduction

Neoadjuvant chemotherapy (NAC) has been increasingly employed as a crucial treatment strategy for breast cancer (1). It aims to reduce tumor volume and downstage the disease, thereby improving surgical resectability and breast-conserving rates. Furthermore, NAC serves as an *in vivo* assessment of tumor sensitivity to chemotherapeutic agents, providing critical information for guiding subsequent treatment decisions. However, patient responses to NAC exhibit significant heterogeneity. While some patients achieve pathological complete response (pCR), which is associated with higher disease-free and overall survival rates (2), others show insensitivity to chemotherapy, with some even experiencing disease progression during treatment (3). Therefore, accurately assessing the response to NAC is essential to avoid unnecessary drug-related toxicity and to allow for timely treatment modification.

The evaluation of the efficacy of NAC in breast cancer mainly relies on pathological and imaging assessments. Pathological evaluation remains the “gold standard” for assessing NAC outcomes (4); however, its major limitation is its retrospective nature, as it cannot provide real-time feedback on treatment effectiveness. In contrast, imaging assessment offers advantages of being non-invasive, reproducible, and capable of dynamic monitoring, allowing for real-time evaluation of tumor response (5, 6). Common imaging modalities include ultrasound (US), magnetic resonance imaging (MRI), and mammography (MM). Among these, breast ultrasound has become the most widely used radiological tool for monitoring NAC efficacy. Ultrasound can evaluate changes in tumor size, morphology, and vascularity, providing valuable information for the early assessment of treatment response. Nevertheless, NAC induces diverse histopathological changes in tumor cells. Some tumors may exhibit a pattern of concentric shrinkage, while others might disintegrate or fragment into scattered islands of tumor cells. In the latter case, although a therapeutic response exists, it may not be fully captured by simple measurements of tumor size, despite the method’s advantages in cost-effectiveness (7). Furthermore, a substantial loss of viable tumor cells may not always be reflected by a reduction in tumor diameter, as the fibrotic stroma can persist (8). Consequently, there is growing research interest in “tumor shrinkage patterns.”

Tumor shrinkage pattern refers to the trend in changes of the overall morphology and three-dimensional volume of the tumor before and after treatment. Imaging assesses these patterns by evaluating changes in the morphology, size, and enhancement of residual lesions after NAC. Although a unified classification system is currently lacking, most studies categorize these patterns into two main types: concentric shrinkage (CS) and non-concentric shrinkage (NCS) (9, 10). MRI-based studies have suggested that early shrinkage patterns during NAC can accurately predict the pathological response in breast cancer. CS is significantly associated with pCR, whereas mixed or NCS patterns are linked to non-pCR. Furthermore, lesions demonstrating CS show a higher rate of tumor size reduction at mid-NAC than those with NCS (11, 12). Candelaria et al. (13) established an ultrasound-based classification system for tumor shrinkage, categorizing responses into “complete,” “concentric,” “fragmented,” “stable,” and “progression.” Their results demonstrated a significant correlation between the mid-NAC ultrasound shrinkage pattern and pCR, with complete and concentric response patterns being more likely to achieve pCR than others. This study confirmed the feasibility of using ultrasound-based shrinkage patterns to predict pCR; however, it was focused solely on triple-negative breast cancer and did not develop a predictive model based on these findings.

Therefore, our study focuses on establishing an ultrasound-based tumor shrinkage pattern for breast cancer under NAC by assessing changes in tumor size, morphology, and internal echogenicity at mid-treatment. Furthermore, we aim to construct a predictive model for treatment response, intending to provide a novel approach for predicting NAC efficacy.

## Materials and methods

### Patient population

A total of 404 female breast cancer patients with 421 breast masses (including 17 patients with bilateral lesions) who presented at Zhangzhou Hospital Affiliated to Fujian Medical University between January 2017 and December 2024 were initially considered for this study. All analyses were performed at the lesion level, as each breast mass was considered an independent unit of observation. The inclusion criteria were as follows: (1) pathologically confirmed invasive breast cancer with available immunohistochemical (IHC) results before NAC; (2) availability of ultrasound examinations performed both before and at mid-NAC; (3) no prior antitumor therapy before initiating NAC; (4) availability of postoperative pathological assessment using the Miller-Payne (M-P) grading system; and (5) lesions presenting as definite masses on baseline ultrasound, enabling reliable morphological assessment.

The exclusion criteria were: (1) failure to complete the full course of NAC; (2) poor-quality ultrasound images precluding reliable evaluation; (3) presence of other concurrent malignancies or distant metastasis; (4) recurrent breast cancer; (5) incomplete clinical or pathological data;and (6) changes in the chemotherapeutic regimen during NAC.

Based on these criteria, 44 cases were excluded for not completing the full NAC regimen, 37 were excluded due to poor-quality ultrasound images, 37 were excluded due to distant metastasis, 5 were excluded due to a prior history of breast disease or tumor recurrence, 5 were excluded because of unavailable Ki-67 expression data, and 6 were excluded due to changes in the chemotherapy regimen. All patients received standard NAC regimens per the National Comprehensive Cancer Network guidelines. Total cycles were 6 or 8, and the mid-treatment ultrasound was performed 21 days after the 3rd cycle (for 6-cycle regimens) or the 4th cycle (for 8-cycle regimens). Consequently, 287 lesions were ultimately included in the study. The age of the enrolled patients ranged from 25 to 73 years, with a median age of 52 (interquartile range: 45, 58.5) years.

This study was approved by Zhangzhou Affiliated Hospital of Fujian Medical University (No. 2025LWB482). Informed consent was waived due to the retrospective nature of the study.

### Ultrasonography examination and evaluation

Breast ultrasound examinations were performed both before NAC initiation and at the mid-NAC time point. The examinations were conducted using ultrasound systems from major manufacturers, including Siemens ACUSON Sequoia, GE Voluson E8, and Mindray Resona 7s, all equipped with linear array transducers (7–15 MHz). With the patient in the supine position and both breasts fully exposed, comprehensive scanning was performed to examine the bilateral breast tissue, axillary regions, and supraclavicular fossae.

To ensure comparability between the pre-treatment and mid-treatment ultrasound assessments, all measurements were consistently obtained on the maximum cross-sectional plane of the lesion at both time points. In cases where the maximum plane was not readily identifiable on the mid-treatment scan due to marked tumor shrinkage, the corresponding plane was determined by referring to anatomical landmarks (e.g., distance from the nipple, depth from the skin, and surrounding vascular structures) to approximate the same section. Two sonographers, each with over ten years of experience in breast ultrasound diagnosis, retrospectively reviewed the images independently. The reviewers were blinded to the patients’ clinical and pathological information. The ultrasound features of the masses were assessed and measured according to the Breast Imaging Reporting and Data System (BI-RADS) classification criteria. The evaluated characteristics included tumor size, morphology, margin, internal echogenicity, presence of liquefaction, changes in deep echogenicity, and Adler grade. The changes in these features between the two time points were documented.

The change in tumor size was quantitatively evaluated using the rate of area change (ΔS), calculated with the following formula: ΔS = (S_m_ - S_0_)/S_0_ × 100%, where S_0_ represents the baseline area before NAC, and S_m_ represents the area measured at mid-NAC. Any discrepancies in assessment between the two reviewers were resolved through discussion until a consensus was reached, ensuring consistency of the final classifications used for analysis.

### Assessment of tumor shrinkage patterns

**Figure 1:**
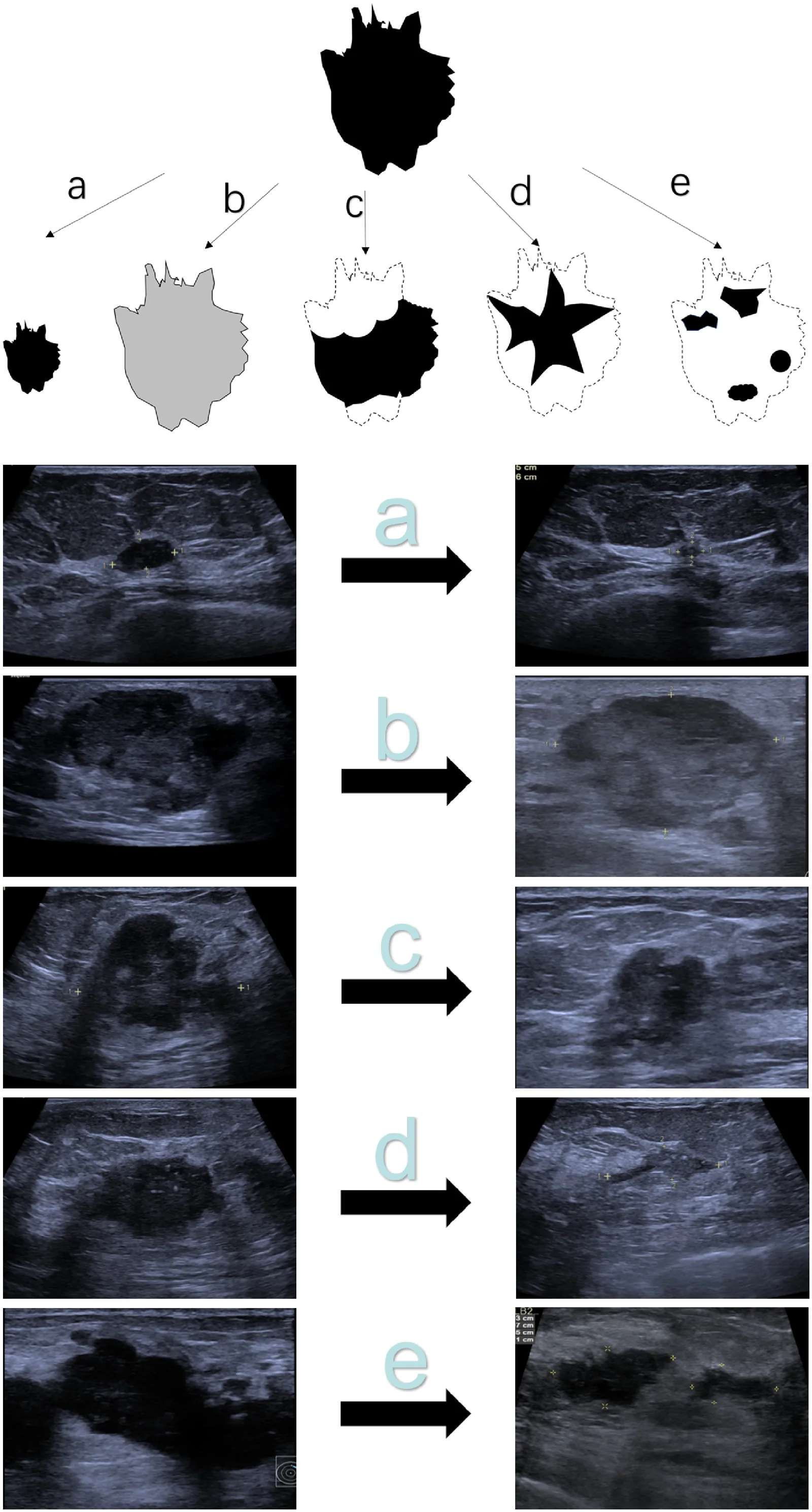
Ultrasound-based tumor shrinkage patterns in breast cancer during neoadjuvant chemotherapy: schematic diagrams and representative real-image cases. (**(A–E)**, upper panel) Schematic diagrams illustrating the five defined shrinkage patterns. **(A)** Concentric shrinkage (CS): homogeneous inward reduction; **(B)** Echogenic change: size stability with internal echo alteration; **(C)** Partial shrinkage: focal marginal retraction with preserved main bulk; **(D)** Dendritic shrinkage: multifocal retraction with spiculated contour; **(E)** Fragmentation: disintegration into scattered nodules. (**(A–E)**, lower panel) Corresponding representative real ultrasound images from five distinct patients, each showing baseline (pre-NAC, left) and mid-NAC (right) paired views.

Based on changes in tumor size, morphology, and internal echogenicity, an ultrasound-based classification of tumor shrinkage patterns was established in this study (**Figure 1**). The patterns were categorized into two primary types: concentric shrinkage (CS) and non-concentric shrinkage (NCS).

CS was defined as a homogeneous, inward reduction of the entire tumor mass toward its center, with no residual scattered cancerous foci in the surrounding tissue (**Figure 1A**).

NCS was characterized by heterogeneous shrinkage of the tumor mass. This category encompassed four distinct sub-patterns:

1. Echogenic Change: Defined as a noticeable alteration in the internal echogenicity of the lesion with little to no change in its overall size or contour (**Figure 1B**).
2. Partial Shrinkage: Characterized by significant, focal shrinkage, predominantly at the tumor margin, while the overall shape and bulk of the original mass are largely retained, presenting an uneven appearance at the edges (**Figure 1C**).
3. Dendritic Shrinkage: Identified when the tumor exhibited multifocal areas of shrinkage, resulting in a spiculated or “dendritic” contour (**Figure 1D**).
4. Fragmentation: Defined as the disintegration of the original tumor into multiple, scattered small nodules within the original tumor bed (**Figure 1E**).

### Histopathological assessment

Pathological specimens obtained from pre-NAC core needle biopsies and post-NAC surgically resected breast masses were processed into sections, routinely stained with hematoxylin and eosin (H&E), and subsequently subjected to immunohistochemical (IHC) staining for diagnosis. The results were independently evaluated by two senior pathologists in a double-blinded manner, following the St. Gallen International Expert Consensus guidelines. Routine diagnostic details were recorded, including tumor size, pathological type, histological grade, the morphological distribution and quantity of residual cancerous foci, IHC findings, and lymph node status. The assessed markers included HER-2 status and the Ki-67 expression rate, among others. The chemotherapy response was evaluated according to the M-P grading system for neoadjuvant chemotherapy.

Using the M-P grading system as the pathological standard for assessing NAC efficacy, post-NAC surgical pathological sections were compared with pre-NAC biopsy sections to evaluate the reduction in tumor cellularity. The response was graded from G1 to G5. Cases achieving an M-P grade of G5 in the primary breast tumor, regardless of axillary nodal status, were classified into the pCR group, while all other cases were classified as non-pCR.

### Statistical analysis

All statistical analyses were performed using R(version 4.3.3). The *Kolmogorov-Smirnov* test was used to assess normality. As all continuous variables were non-normally distributed, they are presented as median (P25, P75), while categorical variables are expressed as percentages (%). Univariate analysis was first conducted to identify potential factors associated with the outcome. Subsequently, multivariate binary *Logistic* regression analysis was performed using a stepwise selection process guided by the Akaike Information Criterion (AIC) to identify independent predictors of pCR. To reduce the risk of overfitting, we employed a 7:3 split into training and testing sets and performed internal validation of the final model. This analysis yielded odds ratios (OR) with 95% confidence intervals (CI) for the independent predictors identified. To account for the potential lack of statistical independence introduced by the 17 patients with bilateral lesions (5.9% of all lesions), we performed a sensitivity analysis by excluding these bilateral cases and re-running the multivariate model. The results (direction and significance of all independent predictors) remained unchanged (data not shown).

A predictive model was constructed based on the results of the multivariate analysis. The model’s discriminatory ability was evaluated by plotting a receiver operating characteristic (ROC) curve and calculating the area under the curve (AUC). The model’s goodness-of-fit was assessed using the *Hosmer-Lemeshow* (H-L) test and visualized with a calibration curve. Finally, decision curve analysis (DCA) was employed to evaluate the clinical utility and net benefit of the predictive model.

## Results

### Patient and baseline characteristics

The age of the enrolled patients ranged from 25 to 73 years, with a median age of 52 (IQR: 45, 58.5) years. The lesions were randomly allocated to a training set and a testing set in a ratio of 7:3. Consequently, the training set comprised 202 lesions, of which 85 achieved pCR and 117 were non-pCR. The testing set consisted of 85 lesions, with 36 in the pCR group and 49 in the non-pCR group.

### Results of univariate analysis

In the training set, univariate analysis revealed that the following variables were significantly different between the pCR and non-pCR groups (all P<0.05): tumor shrinkage pattern, HER-2, echogenicity changes, change in deep echogenicity, Adler decrease, margin changes, Ki-67 and ΔS. Conversely, no statistically significant differences were observed between the groups regarding patient age, clinical stage, or liquefaction changes(all P>0.05). The detailed results are presented in **Table 1**.

**Table 1:** Comparison between the pCR and non-pCR groups in the training set.

Parameter	Non-pCR (n=117)	pCR (n=85)	*P*
Age(year)	51.4 ± 8.89	51.8 ± 9.88	0.770
Clinical stage			
T1	7 (6.0%)	4 (4.7%)	-
T2	79 (68%)	55 (65%)	0.762
T3	13 (11%)	21 (25%)	0.149
T4	18 (15%)	5 (5.9%)	0.370
HER-2			<0.001
Negative	68 (58%)	15 (18%)	
Positive	49 (42%)	70 (82%)	
Shrinkage pattern			<0.001
NCS	95 (81%)	28 (33%)	
CS	22 (19%)	57 (67%)	
Margin changes			<0.001
No	84 (72%)	37 (44%)	
Yes	33 (28%)	48 (56%)	
Echogenicity changes			<0.001
No	91 (78%)	39 (46%)	
Yes	26 (22%)	46 (54%)	
Change in deep echogenicity			0.002
No	97 (83%)	54 (64%)	
Yes	20 (17%)	31 (36%)	
Liquefaction changes			
Increase	25 (21%)	15 (18%)	-
Unchanged	66 (56%)	39 (46%)	0.968
Decrease	26 (22%)	31 (36%)	0.103
Adler decrease			<0.001
No	62 (53%)	15 (18%)	
Yes	55 (47%)	70 (82%)	
Ki-67(%)	30 (20, 50)	50 (35, 60)	<0.001
ΔS [%,M(Q1,Q3)]	-56 (-69, -38)	-78 (-84, -62)	<0.001

### Multivariate *logistic* regression analysis

Variables with a P<0.05 from the univariate analysis comparing the pCR and non-pCR groups (**Table 1**) were included in a multivariate binary *Logistic* regression analysis. The results identified the following factors as independent predictors of pCR (all P< 0.05): CS, ΔS, echogenicity changes, Adler decrease, HER-2 positivity and Ki-67. The detailed results are presented in **Table 2**.

**Table 2:** Multivariate *logistic* regression analysis of predictors for pathological complete response (pCR) in the training set.

Parameter	β	SE	*z*	*P*
X1	1.463	0.442	3.309	<0.001
X2	0.026	0.010	2.490	0.013
X3	1.073	0.432	2.485	0.013
X4	1.448	0.469	2.485	0.002
X5	-0.025	0.011	-2.242	0.025
X6	2.113	0.438	4.824	<0.001
X7	0.602	0.407	1.477	0.140

X1: HER-2; X2: Ki-67; X3: echogenicity changes; X4: Adler decrease; X5: ΔS; X6: shrinkage pattern; X7: Margin changes.

### Construction and performance evaluation of the nomogram model

Variable selection based on the Akaike Information Criterion (AIC) yielded a final multivariate *Logistic* shrinkage model with a minimum AIC value of 171.01. The formula for the model is as follows:


Y = −6.536 + 1.463×X1 + 0.026 × X2 + 1.073 × X3 + 1.448 × X4 − 0.025 × X5 + 2.113 × X6

**Figure 2:**
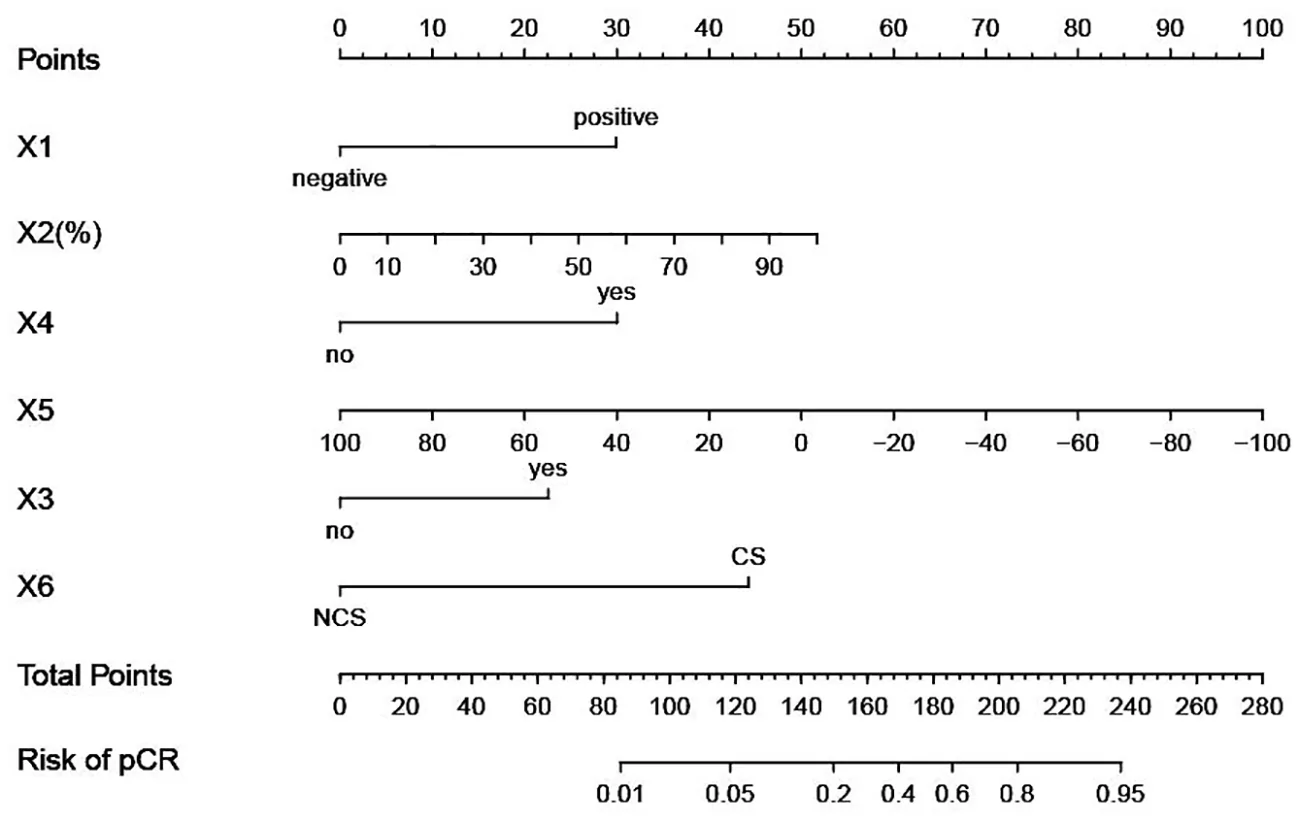
Nomogram for predicting the probability of pathological complete response (pCR) based on independent predictors. X1: HER-2; X2: Ki-67; X3: echogenicity changes; X4: Adler decrease; X5: ΔS; X6: shrinkage pattern.

Based on the independent predictors identified in the multivariate *Logistic* shrinkage analysis, a nomogram for predicting the likelihood of pathological complete response (pCR) was constructed (**Figure 2**). To use the nomogram, the points associated with each patient’s variable values are summed to obtain a total score. This total score is then projected onto the bottom probability scale to estimate the individual patient’s specific probability of achieving pCR.

### Model testing and evaluation

**Figure 3:**
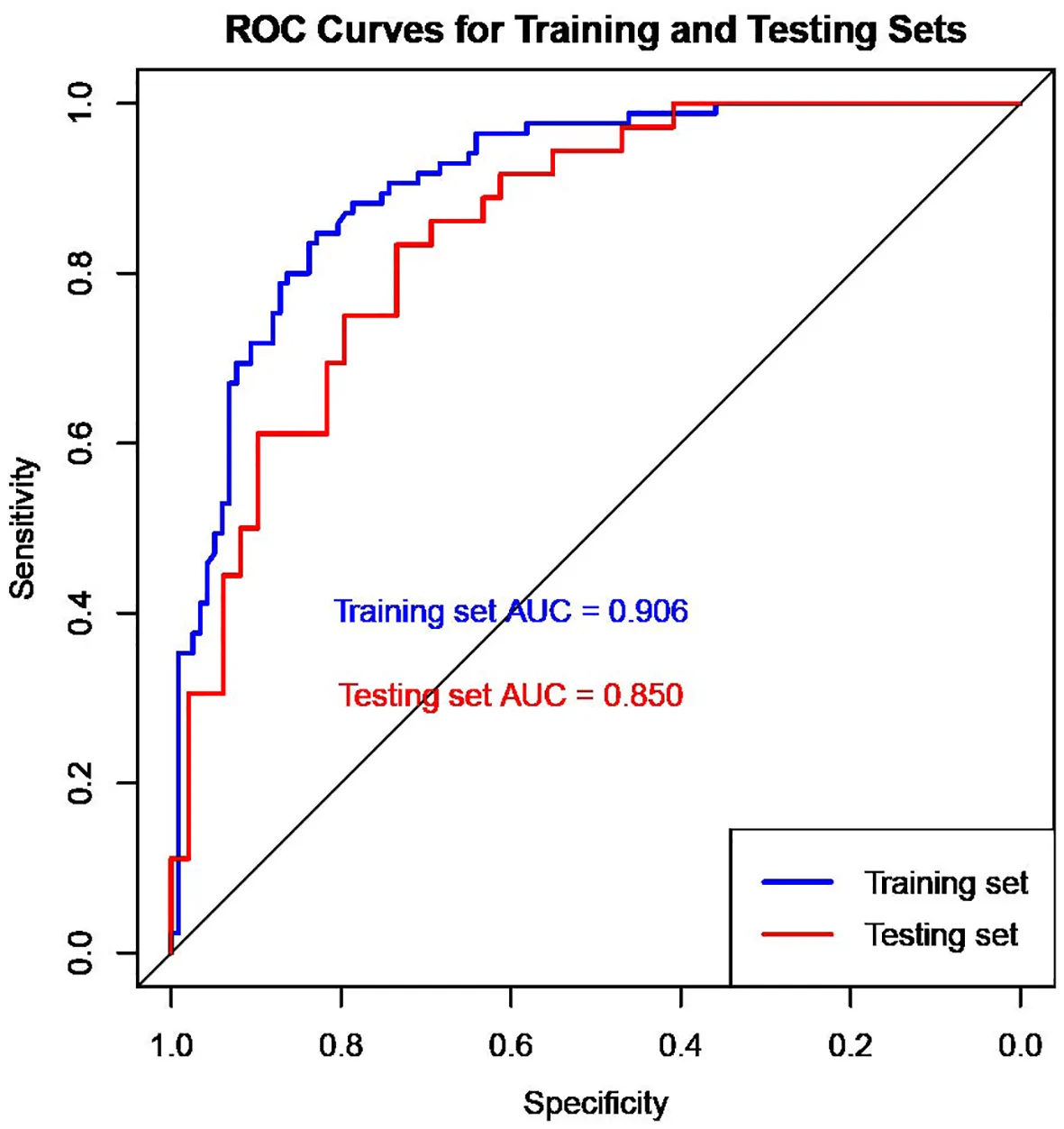
Receiver operating characteristic (ROC) curves of the nomogram.

Receiver operating characteristic (ROC) curves were plotted for both the training and testing sets to assess the discriminatory performance of the nomogram (**Figure 3**). The area under the curve (AUC) was 0.906 (95% CI: 0.866 - 0.946) for the training set and 0.850 (95% CI: 0.770 - 0.929) for the testing set. To evaluate the standalone predictive value of the shrinkage pattern, a univariate logistic regression model using only the shrinkage pattern (CS vs NCS) was constructed, yielding an AUC of 0.741 (95% CI: 0.680 - 0.803).

**Figure 4:**
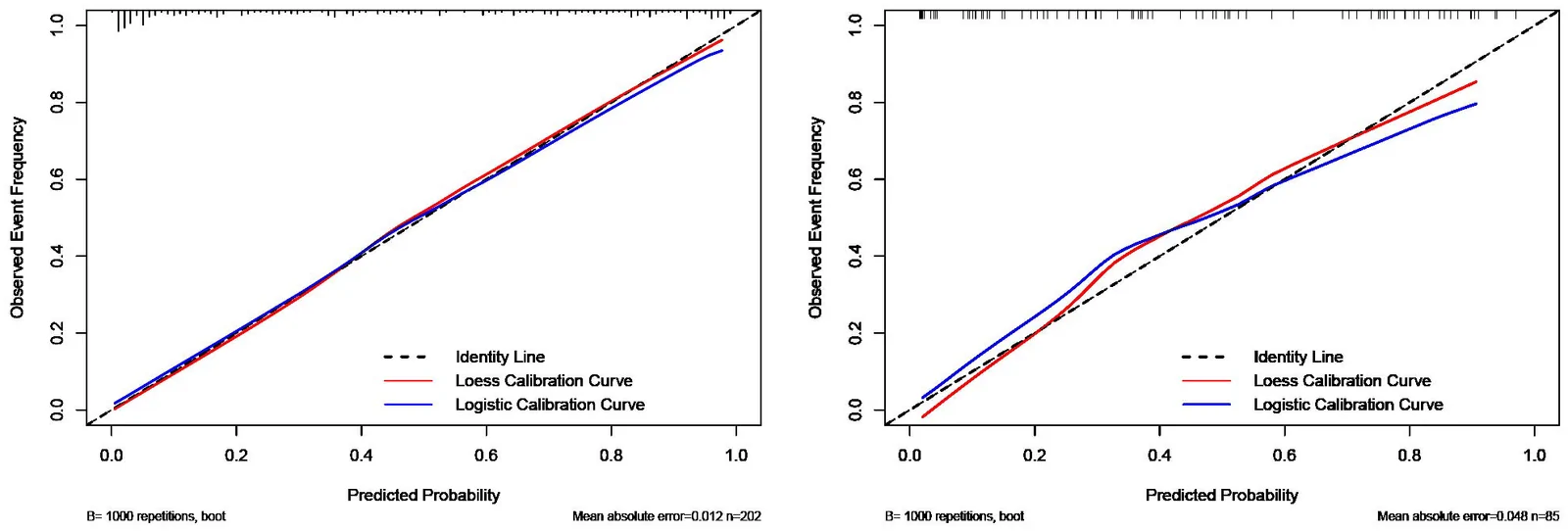
The calibration curves for the training set (left) and test set (right).

The calibration curve results show that the average absolute error of coincidence between the predicted value and the real value is 0.012, and the predicted risk is close to the actual risk, indicating that the predicted coincidence is high (**Figure 4**).

**Figure 5:**
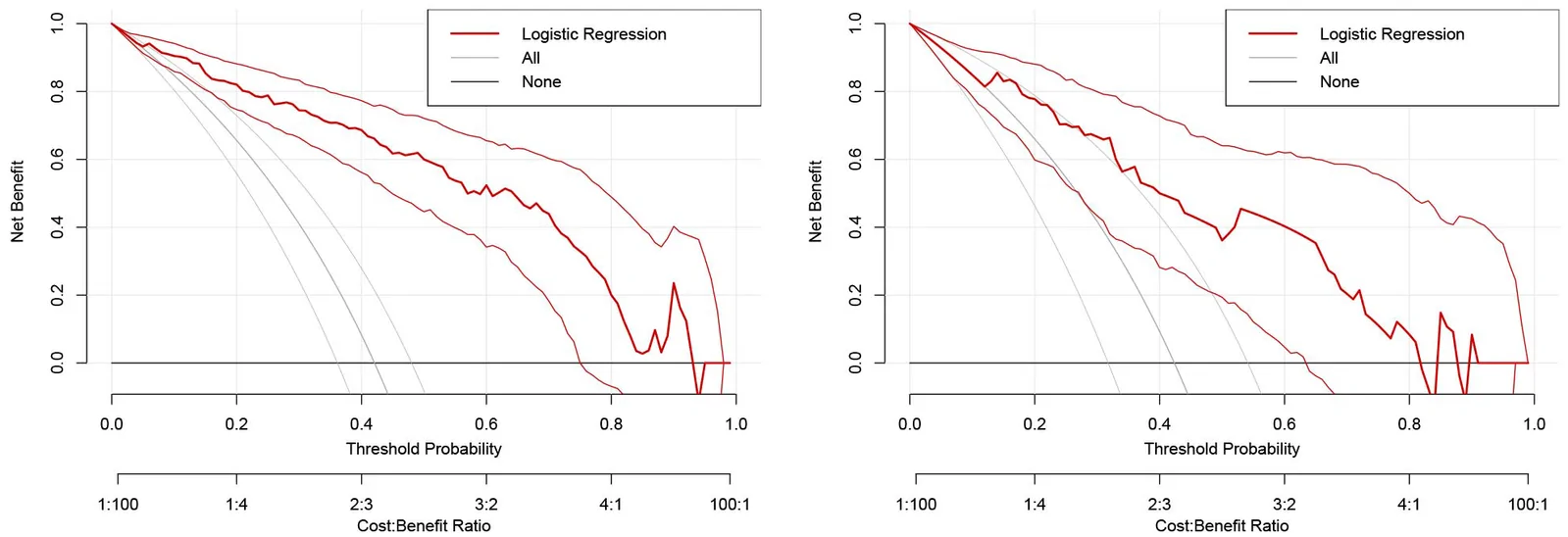
Decision curve analysis (DCA) of the nomogram for predicting pCR in the training (left) and testing (right) sets. The x-axis represents the threshold probability of pCR, and the y-axis represents the net benefit. The red line indicates the nomogram; the light grey diagonal line assumes all patients achieve pCR; the black horizontal line assumes no patients achieve pCR. A greater distance between the red line and the light grey diagonal line indicates a higher clinical net benefit.

Decision curve analysis (DCA) demonstrated that the nomogram provided a net benefit across a wide range of threshold probabilities. The net benefit was observed for threshold probabilities from approximately 0.10 to 0.90 in the training set and from 0.15 to 0.80 in the testing set. The DCA demonstrated that the nomogram provided a positive net benefit across a wide range of threshold probabilities in both the training and testing sets (**Figure 5**).

### Case application

The nomogram enables the prediction of the probability of postoperative pathological response by summing the points assigned to the six variables and locating the total score on the points-to-probability scale. The following two cases illustrate its clinical application:

#### Case 1

**Figure 6:**
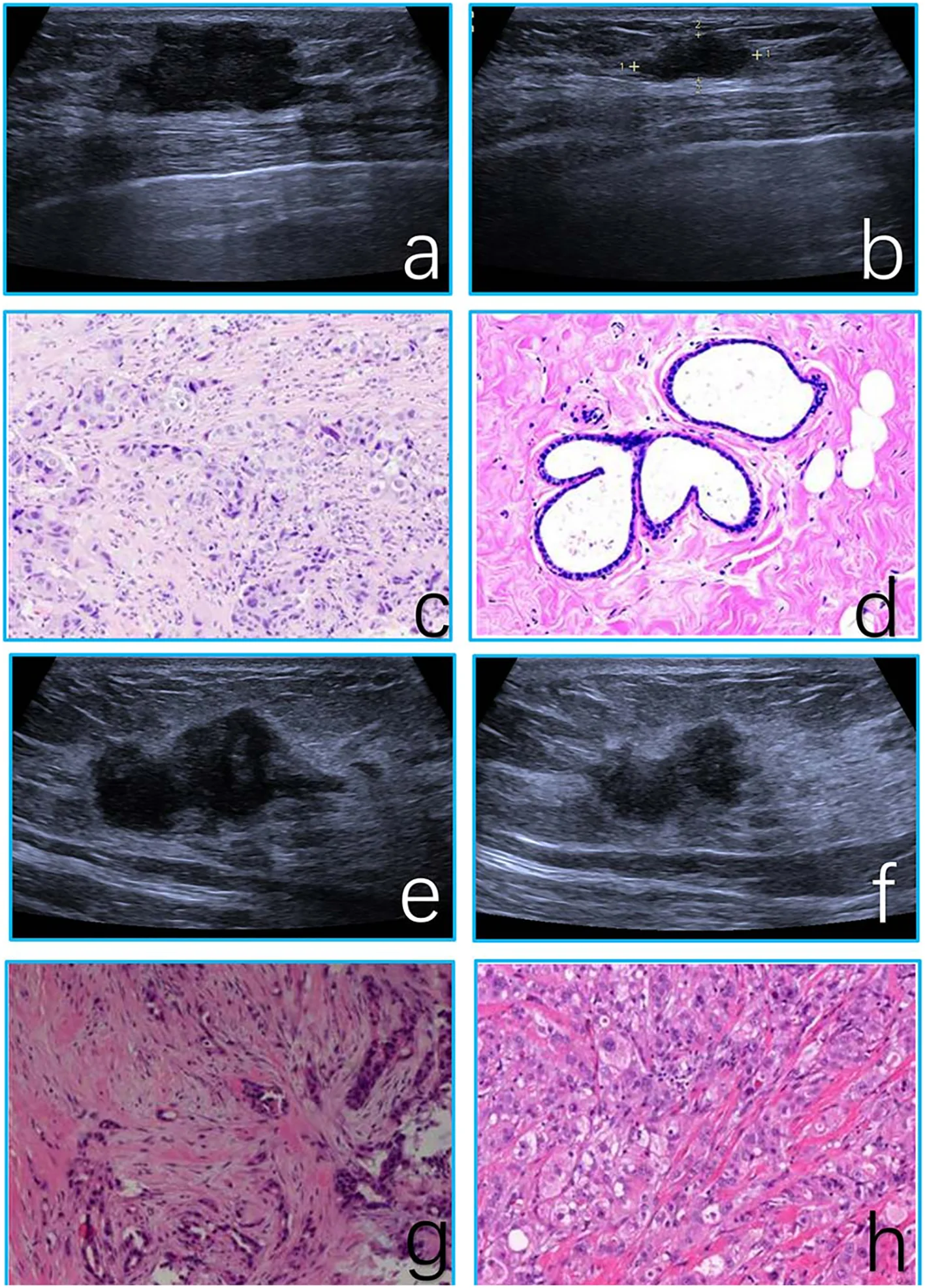
Representative ultrasound images before and during NAC, and corresponding histopathology for two breast cancer cases. **(a–d)** Case 1: a highly sensitive patient, achieving pathological complete response (pCR). **(e–h)** Case 2: a poorly responsive patient with non-pCR outcome. **(a, e)** Pre-NAC ultrasound; **(b, f)** Mid-NAC ultrasound; **(c, g)** Pre-NAC biopsy pathology; **(d, h)** Post-NAC surgical pathology.

Representative ultrasound images obtained before and during the mid-course of NAC are shown in **Figures 6A, B**, respectively. The patient’s scoring profile was as follows: HER-2-positive (30 points), no echogenicity change (0 points), Adler decrease (30 points), CS(44 points), a ΔS value of –74% (87 points), and Ki-67 expression level of 70% (36 points). The total score was 227, corresponding to an estimated pathological complete response (pCR) probability of approximately 90%. Postoperative histopathology confirmed pCR. **Figures 6C, D** show the pre-NAC biopsy pathology and post-NAC surgical pathology.

#### Case 2

**Figure 6E** and 6f illustrate representative ultrasound images of another patient, taken before NAC and at the mid-NAC stage, respectively. The scores for this case were: HER-2-negative (0 points), echogenicity changes(23 points), no Adler decrease(0 points), NCS (0 points), a ΔS value of –44% (72 points), and Ki-67 level of 40% (21 points). The total score was 116, corresponding to a predicted pCR probability of less than 5%. Postoperative pathological evaluation revealed a M-P grade of 3, consistent with a non-pCR outcome. **Figures 6G, H** show the pre-NAC biopsy pathology and post-NAC surgical pathology, respectively.

## Discussion

NAC serves as a crucial treatment strategy for invasive breast cancer, with its efficacy being closely associated with patient prognosis. Studies have demonstrated that patients who achieve pCR exhibit significantly superior disease-free and overall survival rates compared to those who do not (14). Consequently, accurate early prediction of NAC response is vital, as it can help mitigate unnecessary drug-related toxicity, provide clinicians with the opportunity to adjust treatment plans in a timely manner, and ultimately improve patient outcomes and quality of life. The primary objective of our study was to investigate the predictive value of an ultrasound-based tumor shrinkage pattern, identify factors predictive of pCR at mid-NAC, and construct a corresponding predictive model.

This study established an ultrasound-based classification of tumor shrinkage patterns based on changes in sonographic parameters and features at mid-NAC. Notably, our definition of pCR was based solely on the Miller-Payne grading of the primary breast tumor and did not incorporate nodal status, which may overestimate the systemic response rate compared with the standard ypT0/is ypN0 definition. Future studies incorporating both breast and nodal pathological assessment are warranted. Bearing this in mind, we found that the CS pattern was independently associated with pCR, a finding consistent with previous research (12, 13, 15). We also explored the morphological diversity within the NCS category. However, it is important to clarify that the four predefined NCS patterns (echogenic change, partial shrinkage, dendritic shrinkage, and fragmentation) were not strictly mutually exclusive. In many lesions, two or more patterns coexisted, with one being the dominant feature. For example, a tumor might display both echogenic alteration and focal marginal retraction. Consequently, we did not perform a forced reclassification of all NCS cases into single-subtype categories. Instead, we descriptively summarized the predominant patterns and focused our main analysis on the CS versus NCS dichotomy, which demonstrated strong and reproducible predictive value. Studies suggest that tumors exhibiting CS are relatively homogeneous in composition and demonstrate significantly lower tumor heterogeneity compared to those with NCS. This homogeneity likely leads to a more uniform sensitivity to chemotherapeutic agents, making CS tumors more susceptible to achieving pCR following targeted therapy (16). In contrast, residual tumor in NCS patterns is often intermingled with normal breast and adipose tissues, potentially leaving behind scattered cancer cells in areas distant from the tumor center, resulting in non-pCR outcomes (5). Interestingly, we observed that NCS manifests in various sub-patterns, which may be associated with several factors: 1) Lesions with spiculated margins on pre-NAC ultrasound, characterized by distorted local architecture, often result from infiltrative cancer growth beyond the duct wall causing peritumoral fibrosis and interface distortion with normal tissue (17), frequently leading to a “dendritic” shrinkage pattern (**Figure 1D**) post-NAC. 2) Extensive intratumoral fibrosis obscuring the boundary between tumor parenchyma and stroma can cause scattered, irregular distribution of residual cancer foci post-NAC, presenting as a “fragmented” pattern (**Figure 1E**). 3) Furthermore, some studies indicate that clusters of cancer stem cells are more frequently observed and enriched in lesions exhibiting NCS (18), which might be related to the “partial shrinkage” pattern (**Figure 1C**). 4) Clinical practice also reveals that in some breast cancer lesions post-NAC, although the measured dimensions show no significant change, the internal cellular density and composition have already altered (19), corresponding to the “echogenic change” pattern (**Figure 1B**).

Research has confirmed that the rate of tumor size change and alterations in echogenicity between pre- and post-NAC are closely related to pCR outcomes (20, 21). Our study utilized the rate of area change (ΔS) at mid-NAC for assessment. For tumors with NCS, which often undergo irregular morphological changes during shrinkage, ΔS may provide a more reliable reflection of size change than the rate of diameter change (ΔD). The echogenicity of tissue depends on the number and physical properties of scatterers; thus, changes in tumor echogenicity on ultrasound often signify NAC-induced alterations in internal composition. In our study, both ΔS and change in internal echogenicity were independent predictors of pCR, supporting this observation. Additionally, while previous studies suggested a significant reduction in tumor blood flow post-NAC (22), our finding that a reduction in blood perfusion is an independent predictor of pCR is consistent with this conclusion.

HER-2 is a protein that promotes tumor cell growth and proliferation, and the Ki-67 expression rate reflects the proliferative state of breast cancer cells. Tumors that are HER-2-positive or have high Ki-67 expression are highly metabolically active, which may render them more susceptible to NAC drugs. Consistent with previous studies, our study results indicate that patients with HER-2 positivity and higher Ki-67 expression levels often achieve better NAC outcomes (23–26).

In our study, CS, ΔS, echogenicity changes, Adler decrease, HER-2 positivity, and Ki-67 were all identified as independent predictors of pCR (all P< 0.05). The nomogram prediction model constructed based on these factors demonstrated favorable diagnostic performance, with AUC values of 0.906 and 0.850 in the training and testing sets, and accuracy rates of 0.832 and 0.777, respectively. Notably, the shrinkage pattern alone (CS vs NCS) achieved an AUC of 0.741 (95% CI: 0.680 - 0.803), suggesting that while the morphological pattern itself is a moderately strong predictor, its combination with other imaging and pathological parameters provides optimal predictive performance. The Hosmer-Lemeshow test yielded P > 0.05 for both sets, and the calibration curves closely followed the ideal line, indicating adequate model fit and acceptable agreement between the predicted probability of pCR and the actual observed outcomes. Decision curve analysis (DCA) showed that using this nomogram for prediction suggested potential clinical utility in this cohort across a wide range of threshold probabilities.

## Limitations and prospects

This study has several limitations: (1) It was a single-center, retrospective study with a relatively small sample size, which may increase the risk of overfitting, selection bias, and regional bias. Additionally, identical imaging planes across time points could not be ensured, although we used anatomical landmarks and dual-reviewer consensus to mitigate this. Furthermore, the limited sample size precluded a stratified analysis based on different molecular subtypes of invasive breast cancer. (2) The study primarily incorporated ultrasound and immunohistochemical indicators, omitting other potentially relevant markers such as genomic biomarkers or serum metabolic markers. (3) The study did not extend to prognostic and survival analysis for breast cancer patients undergoing NAC. (4) The proposed four NCS subtypes were defined conceptually rather than as mutually exclusive categories. In clinical practice, many lesions exhibited overlapping features, making it challenging to assign a single subtype label. This inherent subjectivity, combined with the small sample size in certain pattern subgroups, precluded formal statistical comparisons between subtypes. We therefore emphasize the CS versus NCS distinction as the primary clinically meaningful classification, and acknowledge that further refinement of NCS subtyping will require larger, prospective cohorts with standardized imaging criteria. Future efforts should focus on conducting multi-center, large-sample, prospective studies to more comprehensively and scientifically explore predictive indicators for NAC response in invasive breast cancer, aiming to develop superior and more comprehensive predictive models to guide clinical practice and improve pCR rates and survival outcomes.

## Conclusion

This study established an ultrasound-based tumor shrinkage pattern at mid-NAC, categorized into CS and NCS, based on changes in tumor size, morphology, and internal echogenicity. The CS pattern, ΔS, echogenicity changes, Adler decrease, HER-2 positivity and Ki-67 were identified as independent predictive factors for pCR at mid-NAC in invasive breast cancer. The constructed nomogram prediction model, incorporating these ultrasound features and clinicopathological factors, demonstrated good diagnostic performance in this retrospective cohort. It may serve as a promising reference tool for predicting NAC outcomes at the mid-treatment stage and could provide useful information to support early treatment decisions. However, prospective multi-center studies with external validation are required before this model can be recommended for routine clinical practice.

## Data Availability

The original contributions presented in the study are included in the article/supplementary material. Further inquiries can be directed to the corresponding author.
